# Scale-Dependent Effects of Grazing on Plant C: N: P Stoichiometry and Linkages to Ecosystem Functioning in the Inner Mongolia Grassland

**DOI:** 10.1371/journal.pone.0051750

**Published:** 2012-12-14

**Authors:** Shuxia Zheng, Haiyan Ren, Wenhuai Li, Zhichun Lan

**Affiliations:** State Key Laboratory of Vegetation and Environmental Change, Institute of Botany, Chinese Academy of Sciences, Beijing, China; Jyväskylä University, Finland

## Abstract

**Background:**

Livestock grazing is the most prevalent land use of grasslands worldwide. The effects of grazing on plant C, N, P contents and stoichiometry across hierarchical levels, however, have rarely been studied; particularly whether the effects are mediated by resource availability and the underpinning mechanisms remain largely unclear.

**Methodology/Principal Findings:**

Using a multi-organization-level approach, we examined the effects of grazing on the C, N, and P contents and stoichiometry in plant tissues (leaves and roots) and linkages to ecosystem functioning across three vegetation types (meadow, meadow steppe, and typical steppe) in the Inner Mongolia grassland, China. Our results showed that the effects of grazing on the C, N, and P contents and stoichiometry in leaves and roots differed substantially among vegetation types and across different hierarchical levels (species, functional group, and vegetation type levels). The magnitude of positive effects of grazing on leaf N and P contents increased progressively along the hierarchy of organizational levels in the meadow, whereas its negative effect on leaf N content decreased considerably along hierarchical levels in both the typical and meadow steppes. Grazing increased N and P allocation to aboveground in the meadow, while greater N and P allocation to belowground was found in the typical and meadow steppes. The differences in soil properties, plant trait-based resource use strategies, tolerance or defense strategies to grazing, and shifts in functional group composition are likely to be the key mechanisms for the observed patterns among vegetation types.

**Conclusions/Significance:**

Our findings suggest that the enhanced vegetation-type-level N contents by grazing and species compensatory feedbacks may be insufficient to prevent widespread declines in primary productivity in the Inner Mongolia grassland. Hence, it is essential to reduce the currently high stocking rates and restore the vast degraded steppes for sustainable development of arid and semiarid grasslands.

## Introduction

Livestock grazing is the most prevalent land-use type of grasslands worldwide, and has the potential to substantially affect community structure and primary productivity [1], [2], alter nutrient cycling [3], [4], [5], [6] and carbon (C) and nitrogen (N) pools [7]–[9] in grasslands. Overgrazing, in particular, has profound effects on multiple ecosystem functions and services, such as N retention, carbon sequestration, biodiversity conservation, and ecosystem stability [10]–[12]. Previous studies have demonstrated that the impacts of herbivores on ecosystem N cycling can range from positive to negative. The accelerating N cycling hypothesis [4], [13]–[15] predicts that herbivore increases the tissue loss of grazing-tolerant species with high N content and litter quality, and directly deposits urine and feces to enhance soil available N, which stimulates net N mineralization and N utilization by plants, leading to the enhancement in shoot nutrients. The decelerating N cycling hypothesis [9], [16], [17], on the contrary, argues that grazing depresses the palatable and N-rich species due to herbivore selectivity, but promotes the dominance of those N-poor or defended species with low litter quality, contributing to slow N turnover and utilization, reducing shoot nutrients. Whether nutrient cycling increases or decreases in response to grazing likely depends on many abiotic and biotic factors, such as resource availability (e.g., soil water and nutrients), grazing duration, herbivore species, plant species and functional group compositions, and plant functional traits [9], [18]–[20].

A functional trait-based approach to community ecology has recently emerged as a promising way to understand plant adaptive strategies, plant-herbivore interactions, and how they link to ecosystem functioning [21]–[23]. The C, N and P contents (%) and stoichiometry in plant tissues (e.g., leaves and roots) are associated with plant growth and ecosystem attributes, which strongly influence ecosystem processes (e.g., N cycling and litter decomposition) and plant responses to various environment variables and disturbance (e.g., precipitation, soil moisture, and grazing pressure) [24]. The N contents of leaves and roots are closely related to plant N uptake and utilization, and indicate N allocation between above- and belowground tissues. Previous studies demonstrated that grazing altered the C and N allocation between plant tissues [9], [25], [26], and heavy grazing increased N allocation to aboveground tissues [27]. Several studies also proposed that plant N and biomass allocation are largely dependent on resource availability [28]–[30]. For example, high soil nutrient availability and low light availability would favor plants allocating more N to stems or leaves than roots [28]. Therefore, grazing-induced shifts in resource allocation (e.g., N and P) between above- and belowground tissues may be meditated by resource availability. The stoichiometric ratios of C: N: P in plant leaves and litter have been widely used to determine N versus P limitation on plant growth, primary productivity and litter decomposition [31]–[34]. In general, plant species can generate positive feedbacks to N cycling, directly through uptake, utilize and loss of nutrients, and indirectly by influencing herbivory selectivity and microbial activity [35], [36]. Hence, plant resource-use strategies, above- vs. belowground competitive abilities, and tolerance vs. defense strategies to grazing may be responsible for diverse stoichiometric responses of different species, which are largely dependent on plant functional traits (e.g., physiological and morphological traits) [21], [23], [37], [38].

Leaf and root traits, e.g., N content of leaves and roots, specific leaf area (SLA), specific root length (SRL), net photosynthetic rate (Pn), photosynthetic nutrient use efficiency (PNUE), and water use efficiency (WUE), reflect plant resource-use strategies (acquisitive vs. conservative) and functional advantage in aboveground or light competition versus belowground competition [21], [28], [38]. The whole plant traits, e.g., plant height, individual biomass, root: shoot ratio, and stem: leaf ratio, reflect plant biomass allocation between tissues and competitive capacity for light [29], [39]. In addition, leaf N content and C: N ratio are closely associated with foliar palatability, which further influence herbivore selectivity and plant tolerance or defense strategies to grazing [9], [17]. Thus, plant functional traits may provide important insights into the mechanisms underpinning plant responses to grazing. In the long-term, shifts in species or functional group compositions are likely to affect ecosystem-level response and nutrient dynamics [18]. Therefore, a variety of mechanisms may operate at different scales or organizational levels, which may involve positive, neutral, and negative effects. For a given system, the response may dependent upon the balance of these mechanisms. For example, the plant community-level response to grazing is likely determined by species-level responses, species abundance, and environment conditions [40]–[42]. The counterbalance between positive and negative feedbacks among vegetation types may affect the regional scale response to grazing. Also, the positive or negative effects may accumulate along the hierarchy of organizational levels. Our recent study demonstrated the effects of grazing on leaf traits are scale-dependant and varied with vegetation types [43]. Therefore, it is difficult to identify the mechanisms based on a single scale study, while an integrated research involving multiple organizational levels is critical for elucidating the underlying mechanisms of grazing impacts on ecosystem properties.

The arid and semiarid grasslands on the Mongolia plateau, which include diverse vegetation types and distribute widely across the Eurasian Steppe region, have been historically subjected to continuous grazing by livestock with high stocking rates, leading to widespread degradation in ecosystem function and services in recent decades [12], [44]. In the Inner Mongolia grassland, plant growth and primary productivity are co-limited by water and N availability [45], thus grazing impacts on plant functional traits and ecosystem functioning are likely mediated by water availability. Our recent studies suggest plant responses to grazing were greater in mesic than dry systems [43], and for a given ecosystem greater responses were found in wet than dry years [37].

In this study, we examined the effects of grazing on the C, N, and P contents and stoichiometry in plant tissues and soil across three vegetation types (meadow, meadow steppe, and typical steppe) along a soil moisture gradient in the Xilin River Basin of Inner Mongolia grassland. Specifically, we address three questions: first, how does grazing affect the C, N, and P contents and stoichiometry in plant tissues (leaves and roots) at different hierarchical levels (plant species, functional group, and vegetation type level) and soil? Second, how do grazing effects on ecosystem functioning (e.g., above- and belowground standing biomass, C, N and P pools, and nutrient limitation) vary across different vegetation types? Third, what are the major mechanisms underpinning different stoichiometric responses across vegetation types in terms of soil properties, plant functional traits, and shifts in functional group compositions? To explore the underlying mechanisms of how grazing affects plant C: N: P stoichiometry and resource allocation across different levels of hierarchy, we hypothesize that: (1) the effects of grazing on plant C, N, and P contents and stoichiometry may become stronger along the hierarchical levels, i.e., from species to functional group to vegetation type. (2) The grazing effects on plant C: N: P stoichiometry and N and P allocation may strongly depend on resource availability, such as water and N. We expect that grazing may have positive effects on leaf nutrients (e.g., N and P contents) but negative effects on root nutrients in the wet and fertile meadow. In contrast, grazing may have no effects or even negative effects on leaf nutrients but positive effects on root nutrients in the dry and infertile typical steppe. Hence, grazing may elevate N and P allocation to aboveground in the meadow, but it may enhance N and P allocation to belowground in the typical and meadow steppes.

## Methods

### Study Area

The study was conducted in the Xilin River Basin (43°26′–44°29′N, 115°32′–117°12′E), which is located in the typical steppe region of the Inner Mongolia grassland, northern China, and covers an area of 10 786 km^2^, with elevation ranging from 983 to 1469 m. Mean annual temperature is 0.4°C, with the lowest mean monthly temperature −21.4°C in January and the highest 19.0°C in July. Mean annual precipitation is 336.9 mm yr^−1^, with about 80% occurring in the growing season (May–August). The dominant soil types are typical chestnut and dark chestnut, while meadow soil is a non-zonal soil type in this region [46]. Six pairs of parallel ungrazed and grazed plant communities, i.e., *Carex appendiculata* meadow, *Stipa baicalensis* meadow steppe, *Leymus chinensis* typical steppe, *S*. *grandis* typical steppe, *Caragana microphylla* typical steppe, and *Artemisia frigida* typical steppe were selected along a soil moisture gradient in the Xilin River Basin. The six pairs of plant communities include one for meadow, one for meadow steppe, and four for typical steppe, with the number of sites being proportional to its area. These communities are subjected to similar climatic conditions, such as temperature and precipitation, but differ in terms of floristic composition and soil properties, particularly soil moisture and nutrients. The ungrazed sites of communities are the permanent field sites of the Inner Mongolia Grassland Ecosystem Research Station (IMGERS), Chinese Academy of Sciences, which have been fenced from grazing for about 20–30 years [42]. In contrast, the grazed sites, located outside the fence of ungrazed sites, have been managed as free grazing pasture (mainly by sheep) since 1950s, thus they have about 60 years of grazing history. More detailed information for the six pairs of communities could be available from Zheng et al. [43].

### Field Sampling and Measurements

Field sampling was carried out during 28 July to 14 August, 2007, when the aboveground standing biomass reached its annual peak [42]. At each site, aboveground biomass of plants were sampled by 5–10 quadrats (1×1 m each) located randomly within an area of 100 m ×100 m. Ten quadrats were used for meadow steppe and typical steppe, and 5 quadrats were for the more homogeneous meadow community. For the grazed sites, these quadrats were randomly located in the areas that were not subjected to grazing during the current growing season. Within each quadrat, all living biomass and current year dead materials were harvested by clipping to the soil surface, separated to species, and oven dried at 70°C for 24 h to constant mass and weighed. Litter biomass within each quadrat was collected. The aboveground biomass of each species was collected and transported to a laboratory for stem and leaf separation, then they were oven-dried at 70°C for 24 h to constant mass, thus stem biomass, leaf biomass, and plant biomass could be calculated. The total number of species, number of individuals, and aboveground biomass of each species were measured within each quadrat, which was used for estimating species richness, species abundance, and aboveground standing biomass of community at each site. The relative abundance of each species was obtained by calculating the proportion of individual density of each species to the total density. The relative biomass of each species was determined by its biomass ratio to the total community biomass. Belowground biomass was determined in late August, 2007, when it reached the annual peak in the Inner Mongolia grassland. Belowground biomass was sampled by randomly taking two 7-cm diameter soil cores from 0–20 cm depths inside each quadrat, and totally 10–20 samples at each site. Soil was rinsed out from roots under running water over a 1-mm screen, dead materials were picked out, and then root biomass was oven-dried at 65°C and weighed. Within each quadrat, soil samples were collected by taking three 5-cm diameter soil cores from 0–20 cm depths, mixed in situ as one composite sample, and hand-sorted to remove plant materials. Each soil sample was sieved through 2 mm mesh and separated into two parts. One was air-dried and ground to 80-mesh to determine soil total N, P, and organic C contents, the other maintained fresh to measure soil ammonium (NH_4_^+^–N) and nitrate (NO_3_^−^–N) contents. Soil samples were also taken from 0–20 cm layer with a soil bulk density auger, oven-dried at 105°C for 48 h, and weighed to determine soil bulk density.

After vegetation survey and soil sampling, leaf samples of dominant and common species were collected at each site. For each species, 10–20 fully grown individuals were randomly selected, and 2–3 mature fully expanded leaves were picked, which were further divided into 5 samples. The same number of individuals of each species that were not suffering from grazer bites was also collected at the grazed sites. In this study, 169 shared species at the ungrazed and grazed sites, including 81 genera and 29 families were collected across three vegetation types, that is, 55 species in the meadow, 54 species in the meadow steppe, and 60 species in four communities of the typical steppe. All species were classified into five functional groups based on life forms, i.e., perennial bunchgrasses (PB), perennial rhizome grasses (PR), perennial forbs (PF), annuals and biennials (AB), and shrubs and semi-shrubs (SS). Root samples of 19 shared dominant species were collected at the ungrazed and grazed sites in the meadow (6 species), meadow steppe (6 species) and typical steppe (7 species). For each species, 10 individuals were randomly selected, and root biomass were sampled by taking 7-cm diameter soil cores from 0–20 cm depths after removing aboveground parts, which were further divided into 5 samples. Leaf and root samples were dried at 60°C for 24 h in a forced-draught oven and ground to homogeneity with a ball mill (MM 2000; Retsch GmbH & Co, Haan, Germany) for C, N and P measurements.

The total N, P, and organic C contents (percentage dry mass, %) in soil and plant tissues (leaves and roots) were analyzed using the standard methods [47]. The organic C content was analyzed using K_2_Cr_2_O_7_-H_2_SO_4_ oxidation method. The total N content was determined using the Kjeldahl acid-digestion method with an Alpkem autoanalyzer (Kjektec System 1026 Distilling Unit, Sweden), and total P content was analyzed using the molybdenum blue colorimetric method with a UV/visible spectrophotometer (Beckman Coulter DU 800, USA). Contents of soil NH_4_^+^–N and NO_3_^−^–N (mg kg^−1^) were determined using the 2 mol L^−1^ KCl extraction method (solution: sediment = 5∶1, 1-h extraction, solid separation by centrifugation followed by Whatman #1 paper filtration) with a flow injection autoanalyzer (FIAstar 5000 Analyzer, Foss Tecator, Denmark). Total available soil N was the sum of ammonium and nitrate contents. The total amounts of C, N and P (g m^−2^) in aboveground leaf biomass were calculated by multiplying leaf C, N and P contents with leaf biomass of each species within the quadrat. The C, N and P pool of belowground biomass (0–20 cm, g m^−2^) were calculated from root C, N and P contents multiplied by belowground biomass from 0–20 cm soil depth. Soil C, N and P pool (0–20 cm, g m^−2^) were obtained from the contents multiplied by soil bulk density [9].

### Plant Functional Traits

Plant functional traits, including whole plant traits, e.g., plant height, individual biomass, stem: leaf biomass ratio (SLR), and root: shoot biomass ratio (RSR); leaf traits, e.g., leaf N content, leaf C:N ratio, specific leaf area (SLA), net photosynthetic rate (Pn), photosynthetic nutrient use efficiency (PNUE), and foliar stable carbon isotope composition (*δ* ^13^C); root traits, e.g., root N content, root C:N ratio, and specific root length (SRL); and reproductive traits, e.g., reproductive allocation (fruit biomass ratio) and seed mass were measured for dominant and common species at the ungrazed sites across three vegetation types. In particular, Pn, PNUE, foliar *δ* ^13^C, SRL, and reproductive traits were determined for species only in typical steppe. These plant traits were measured on 10–20 individuals for each species in early August. Plant height was measured by the distance from the basal stem to the natural crown of each individual. After the height measurement, aboveground part of each individual was collected and taken back to the laboratory for stem and leaf separation. All leaves of an individual were picked to determine the projected leaf area with a portable leaf area meter (Li-3100C; Li-COR, Lincoln, NE, USA), and the number of leaves were recorded isochronously. Then the stem and leaf samples were oven-dried at 70°C for 24 h to constant mass and weighted. Hence, individual leaf area, dry mass per leaf, stem biomass, leaf biomass, and individual biomass could be calculated, and specific leaf area (SLA, cm^2^ g^−1^) and stem: leaf ratio (SLR) were separately calculated as the ratio of leaf area to dry mass, and ratio of stem biomass to leaf biomass. The net photosynthetic rate (Pn) was measured with a Li-6400 portable photosynthetic system (Li-6400, Li-Cor, Lincoln, NE, USA) at a CO_2_ concentration of 400 µmol mol^−1^ (using the built-in Li-Cor 6400 CO_2_ controller) and a saturating irradiance of 1500 µmol m^−2^ s^−1^ provided by a built-in red LED light source. The photosynthetic nutrient use efficiency (PNUE, µmol CO_2_ mol^−1^ s^−1^) was defined to equal Pn divided by leaf N content. The foliar *δ* ^13^C value was analyzed with a stable isotope ratio mass spectrometer (MAT-253; Finnigan, San Jose, USA). The root: shoot ratio (RSR) was obtained by calculating the ratio of root biomass to aboveground biomass of each individual. The specific root length (SRL, m g^−1^) was determined by the ratio of total length of third-order roots to its dry mass [48], and root length was analyzed with a root analysis system (Delta-T Scan, Cambridge, UK). The reproductive allocation was calculated as the ratio of fruit biomass to individual biomass. The mature seeds were collected from 10–20 individuals of each species, and the sampling time was determined based on the mature season of species. Seeds were dried at room temperature for a week. For each species, the mean seed mass (mg seed^−1^) was the average dry mass of 20–100 individual seeds.

### Statistical Analysis

Statistical analyses were performed using SAS Version 9.2 (SAS Institute, Cary, North Carolina, USA, 2003). The effects of grazing on C, N, and P contents and C: N: P stoichiometory in soil and plant tissues (leaves and roots) at the species, plant functional group, and vegetation levels were tested with ANOVA, using treatment (grazed and ungrazed), vegetation type (meadow, meadow steppe, and typical steppe ), and all interactions as fixed-effects. A total of 169 species present at the paired ungrazed and grazed sites across three vegetation types were classified into three response groups, i.e., decreased, increased, and unchanged, based on their leaf nutrients response to grazing. The effects of grazing on the C, N and P pools of above- and belowground biomass and soil, above- and belowground standing biomass, and relative aboveground biomass of plant functional groups were also examined across three vegetation types. One-way ANOVAs followed by LSD multiple-range tests were performed to test differences in functional traits among four plant functional groups (PB, PR, PF and AB).

## Results

### Grazing Effects on Leaf Nutrients at Different Levels of Hierarchy

**Figure 1:**
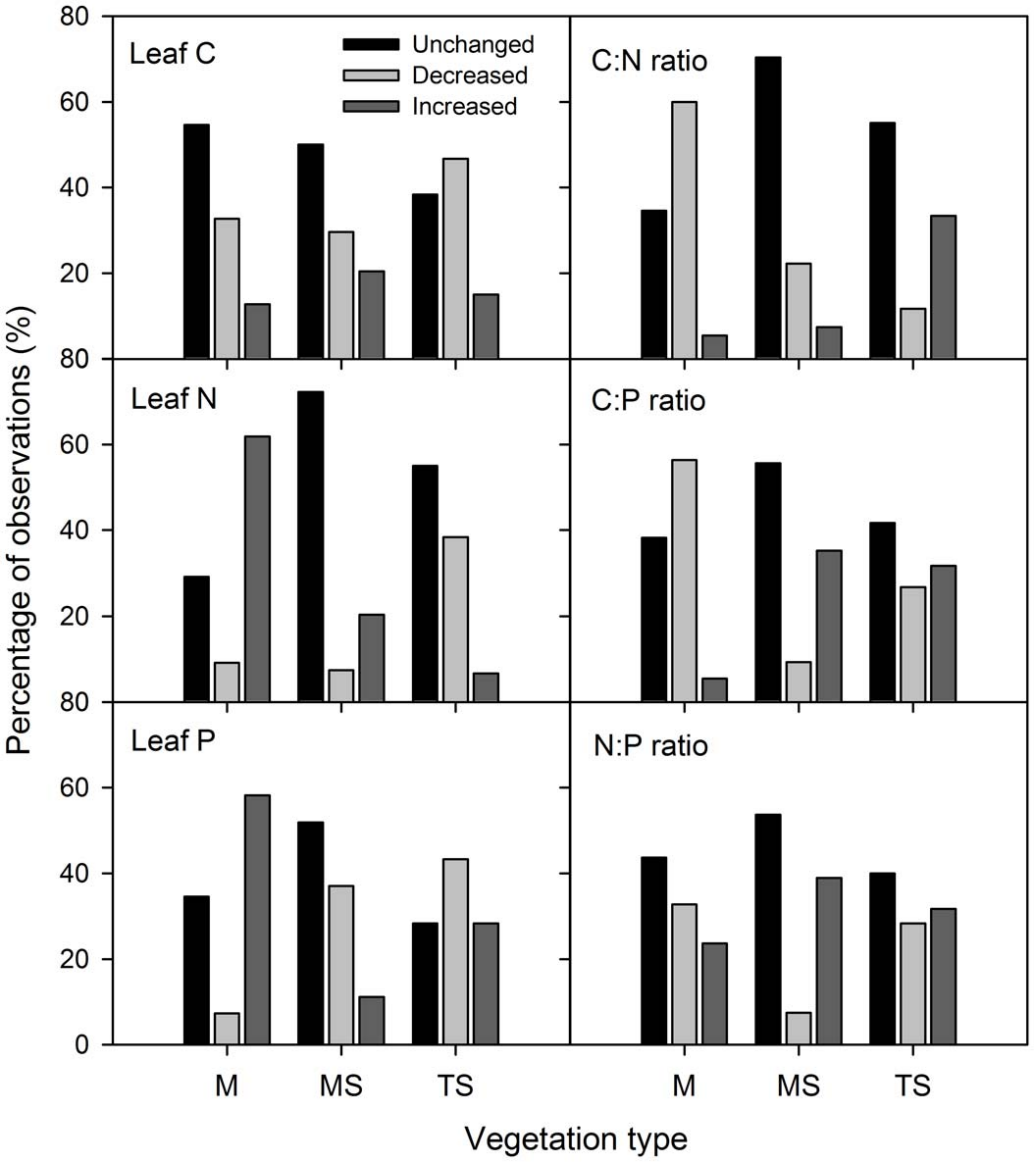
Percentages of species categorized as three groups based on responses of leaf C, N, P contents and stoichiometory to grazing. M, meadow; MS, meadow steppe; and TS, typical steppe. Significant differences (*P*<0.05) between the grazed and ungrazed sites were analyzed for increased, decreased and unchanged response groups.

We examined the responses of leaf C, N and P contents and stoichiometry to grazing in 169 shared species at the ungrazed and grazed sites across three vegetation types. At plant species level, grazing significantly increased leaf N and P contents in 62% and 58% of the total species in the meadow, but leaf C content remained unchanged in 55% of species, resulting in reduction in C: N and C: P ratios in most of the species (Fig. 1). Leaf N: P ratio in 33% of species was diminished by grazing, and that in 44% of species was unchanged. In the meadow steppe, leaf C, N, P contents and stoichiometry remained unchanged in more than 50% of species, with other species either increased or decreased in leaf N and P contents and stoichiometry. In the typical steppe, grazing decreased leaf C, N and P contents in 47%, 38%, and 43% of species, respectively; but had no significant effects on them in 38%, 55% and 28% of species. The ratios of C: N, C: P and N: P increased in 32–33% of species, but they remained unchanged in 40–55% of species.

**Figure 2:**
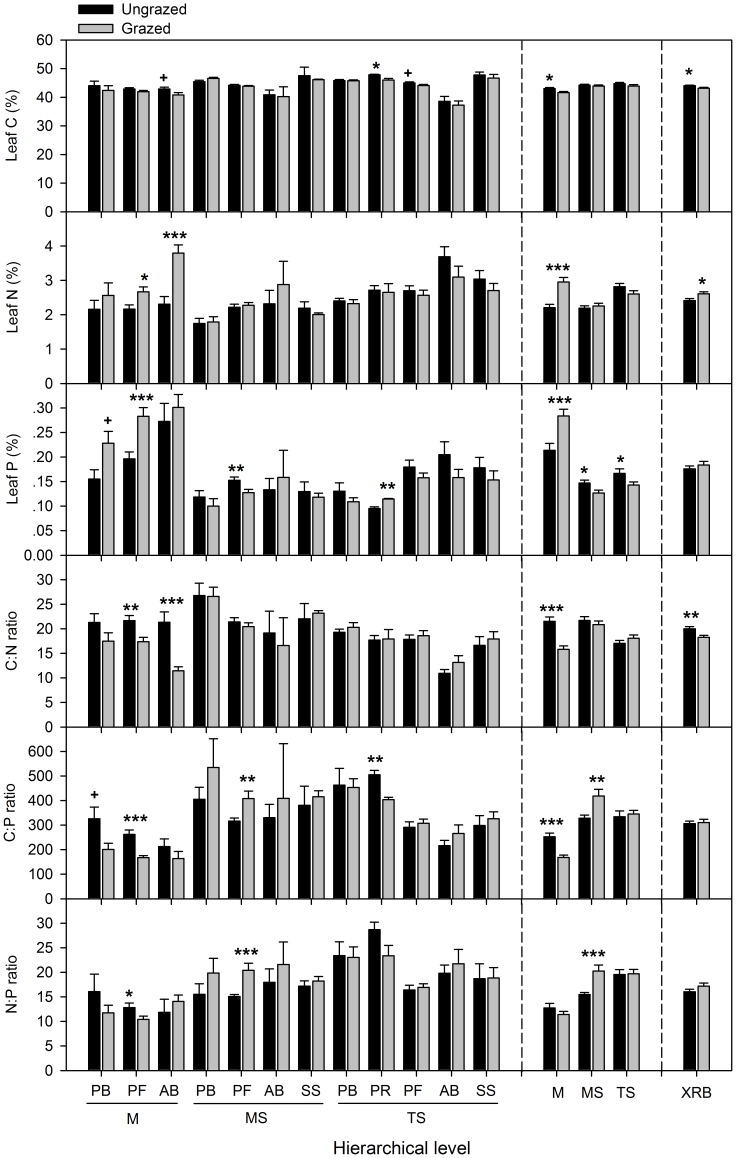
Effects of grazing on leaf C, N, P contents and stoichiometory at plant functional group and vegetation type levels. The error bars are mean+SE. PB, perennial bunchgrasses; PR, perennial rhizomatous grasses; PF, perennial forbs; AB, annuals and biennials; SS, shrubs and semi-shrubs; M, meadow; MS, meadow steppe; TS, typical steppe; and XRB, Xilin River Basin. Significant differences between the grazed and ungrazed sites are reported from ANOVA as +, 0.05<*P*<0.1; *, *P*<0.05; **, *P*<0.01; ***, *P*<0.001.

At functional group level, grazing had larger impacts on the meadow than the meadow steppe and typical steppe. The magnitudes of changes in leaf nutrients and stoichiometry were generally greater in perennial forbs (PF), annuals and biennials (AB), and perennial rhizome grasses (PR) than in perennial bunchgrasses (PB) and shrubs and semi-shrubs (SS) (Fig. 2). In the meadow, leaf N and P contents were significantly increased by grazing, while leaf C content remained unchanged in PF, leading to reduction in C: N, C: P and N: P ratios. For AB, leaf N content was significantly increased, leaf C content was slightly reduced, and leaf P content remained unchanged by grazing, resulting in decreased C: N and unchanged C: P and N: P ratios. For PB, grazing slightly increased leaf P content, and leaf C and N contents remained unchanged, with no change in C: N and N: P and reduction in C: P. In the meadow steppe, grazing diminished only leaf P content in PF and had no effects on leaf C and N contents, resulting in no change in C: N but increases in C: P and N: P ratios. In the typical steppe, leaf N content remained unchanged in all functional groups, leaf P content was increased in PR, and leaf C content was decreased in PB and PR, leading to no change in C: N and N: P ratios but reduced C: P ratio.

At vegetation type level, grazing had greater effects on the meadow than the meadow steppe and typical steppe. In the meadow, both leaf N and P contents were increased, while leaf C content was decreased by grazing, causing reduction in C: N and C: P ratios and no change in N: P ratio (Fig. 2). In the meadow steppe, grazing diminished leaf P, and leaf C and N contents remained unchanged, leading to no change in C: N ratio but increases in C: P and N: P ratios. In the typical steppe, grazing only decreased leaf P content, but had no significant effects on C and N contents and C:N:P stoichiometry. When three vegetation types were pooled together, leaf N was significantly enhanced, leaf C was diminished, and leaf P remained unchanged, leading to diminished C: N ratio in the Xilin River Basin (Fig. 2).

### Root Nutrients and Stoichiometry

At plant species level, grazing significantly enhanced root N in one of six species in the meadow, diminished root C in three species, resulting in decreased C: N ratio in two species, and increased N: P ratio in one species (Fig. S1). Root P and C: P ratio remained unchanged in all six species. In the meadow steppe, grazing increased root N in two of six species and root P in one species, leading to decreased C: P and N: P ratios of this species. In the typical steppe, grazing significantly increased root N in three and P content in four of seven species, but it decreased root C in three species, contributing to the reduced C: N, C: P and N: P ratios in these species.

**Figure 3:**
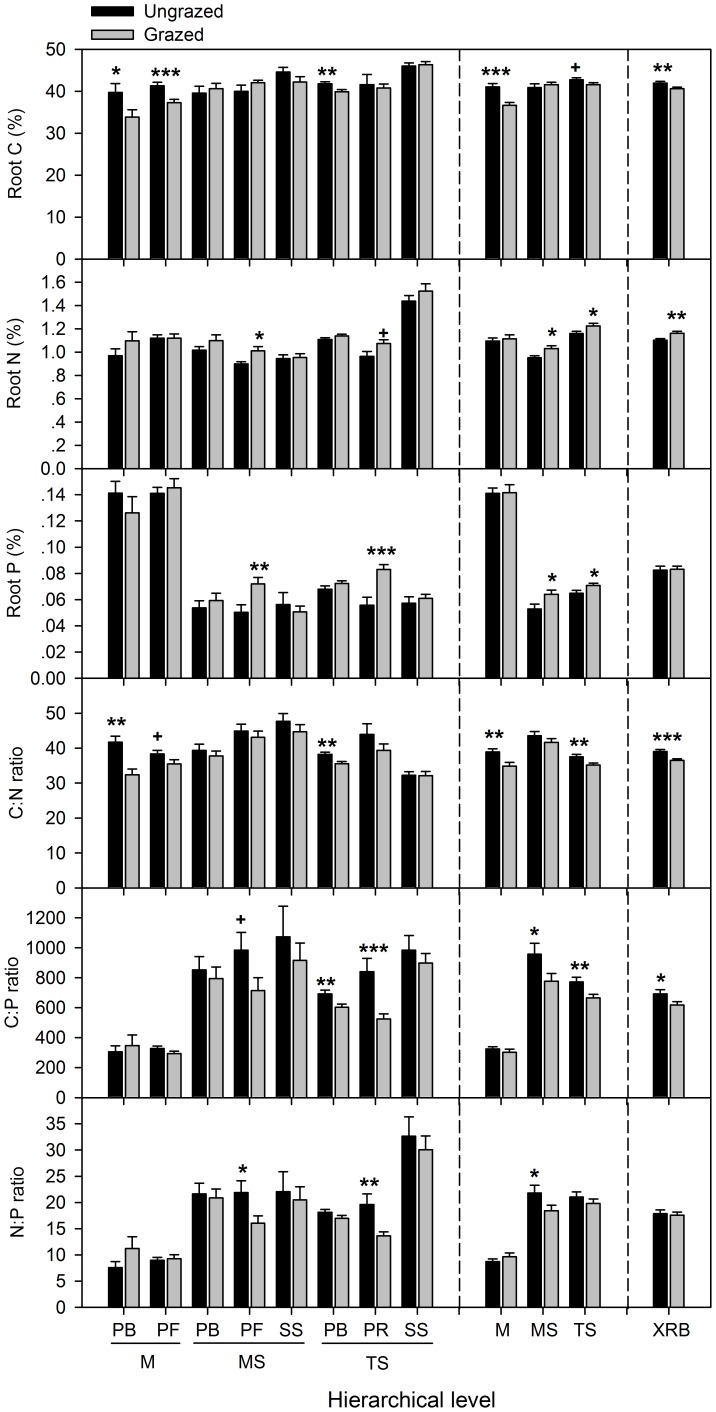
Effects of grazing on root C, N, P contents and stoichiometory at plant functional group and vegetation type levels. The error bars are mean+SE. Significant levels and all symbols are derived as in Fig. 2.

At functional group level, grazing generally had greater effects on PF and PR than PB and SS (Fig. 3). In the meadow, grazing decreased root C content and C: N ratio in PF and PB, but had no significant effects on their N and P contents, C: P and N: P ratios. In the meadow steppe, however, grazing only increased root N and P contents in PF, leading to decreased C: P and N: P ratios. In the typical steppe, grazing increased root N and P contents, but decreased C: P and N: P ratios in PR. For PB, however, grazing decreased root C content, while N and P contents remained unchanged, resulting in declines in root C: N and C: P ratios.

At vegetation type level, grazing had greater effects on the typical steppe than the meadow steppe and meadow (Fig. 3). Root C and C: N ratio decreased while N and P contents and C: P and N: P ratios remained unchanged in the meadow. Grazing significantly increased root N and P contents, while root C content remained unchanged in the meadow steppe, leading to reduction in root C: P and N: P ratios. In the typical steppe, root N and P contents were increased, while root C content was slightly decreased by grazing, which leads to decreases in root C: N and C: P ratios and no change in N: P ratio. In the Xilin River Basin, root N content was significantly increased, root C content was decreased, while root P content remained unchanged, resulting in decreased C: N and C: P ratios and unchanged N: P ratio.

### Soil Nutrients and Stoichiometry

Soil C, N and P contents and total available N (ammonium+nitrate) were much higher in the meadow than the meadow steppe and typical steppe (Fig. S2). Grazing only caused minor shifts in soil nutrients and stoichiometry across the three vegetation types in the Xilin River Basin. Soil P was slightly decreased (*P*<0.1) by grazing in the meadow. Soil C: N was slightly increased (*P*<0.1) in the meadow steppe but decreased in the typical steppe.

### C, N and P Pools of Above- and Belowground

**Figure 4:**
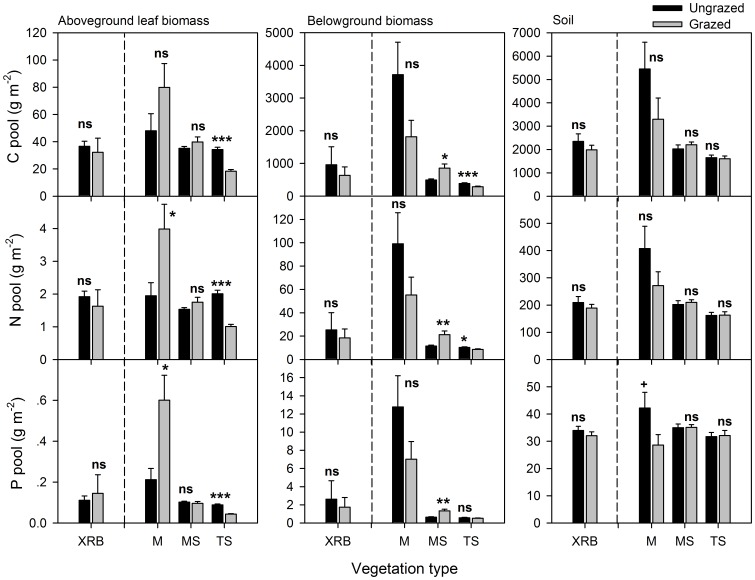
Effects of grazing on C, N, and P pools in above- and belowground biomass and soil (0–20 cm). The error bars are mean+SE. Significant differences between the grazed and ungrazed sites are reported from ANOVA as ns, *P*>0.1; +, 0.05<*P*<0.1; *, *P*<0.05; **, *P*<0.01; ***, *P*<0.001. All symbols are derived as in Fig. 2.

In the meadow, grazing increased the N and P pools of leaf biomass by 104–183%, but had no significant effects on C, N and P pools of belowground biomass due to large variations (Fig. 4). In the meadow steppe, however, grazing significantly increased C, N and P pools of belowground biomass by 74–107%, but had no significant effects on those of aboveground biomass. In the typical steppe, grazing decreased the C, N and P pools of both above- (by 46–51%) and belowground biomass (by 17–25%, except for P pool). Grazing had no significant effects on soil C, N and P pools across the three vegetation types, except soil P pool was slightly decreased (*P*<0.1) in the meadow (Fig. 4). In the Xilin River Basin, the C, N and P pools in above- and belowground biomass and soil remained unchanged.

### Above- and Belowground Standing Biomass and Functional Group Composition

**Figure 5:**
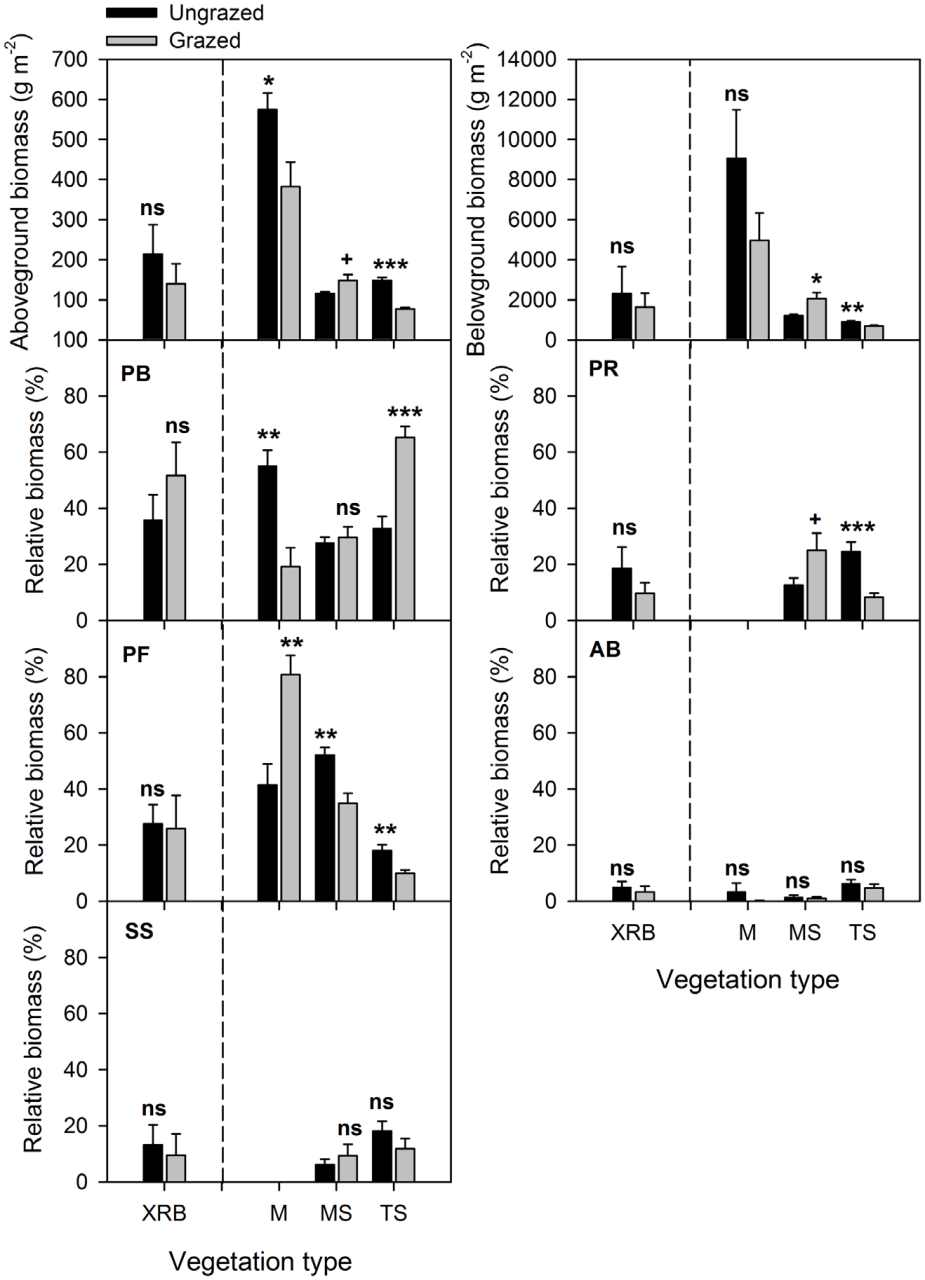
Effects of grazing on above- and belowground standing biomass and plant functional group composition. The error bars are mean+SE. PB, perennial bunchgrasses; PR, perennial rhizomatous grasses; PF, perennial forbs; AB, annuals and biennials; SS, shrubs and semi-shrubs; M, meadow; MS, meadow steppe; TS, typical steppe; and XRB, Xilin River Basin. Significant levels are as in Fig. 4.

Grazing significantly decreased the aboveground standing biomass by 34%, but had no significant effect on belowground biomass in the meadow (Fig. 5). In the meadow steppe, however, belowground biomass was enhanced by 71%, but aboveground biomass remained unchanged. Grazing diminished the aboveground biomass by 48% and belowground biomass by 23% in the typical steppe. On average, both the above- and belowground standing biomass were unchanged in the Xilin River Basin. In the meadow, the relative biomass of PF was increased by 95%, while that of PB was decreased by 65% (Fig. 5). In the meadow steppe, grazing increased the relative biomass of PR by 99%, but decreased that of PB by 33%. In the typical steppe, grazing increased the relative biomass of PB by 98%, but decreased PR by 66% and PF by 45%. The relative biomass of AB and SS, in contrast, remained unchanged across three vegetation types.

### Plant Functional Traits

Multi-trait comparison showed that different functional groups differed substantially (*P*<0.05) in 9 out of 15 plant functional traits, including whole plant traits, leaf traits, root traits, and reproductive traits (Table 1). The PB exhibited greater plant height, individual biomass, root: shoot ratio, stem: leaf ratio, leaf C: N ratio, and root N content, but lower leaf N content, specific leaf area (SLA), root C: N ratio, and reproductive allocation. In contrast, AB showed lower plant height, individual biomass, root: shoot ratio, stem: leaf ratio, leaf C: N ratio, and root N content, but higher leaf N content, SLA, root C: N ratio, and reproductive allocation. Moreover, AB generally had higher net photosynthetic rate (Pn), photosynthetic nutrient use efficiency (PNUE), and water use efficiency indicated by *δ* ^13^C value. For PF and PR (i.e., *Leymus chinensis*), the values of functional traits were intermediate between PB and AB. For example, these species showed relatively lower leaf N content, SLA, Pn, and reproductive allocation, but higher plant height, individual biomass, leaf C: N ratio, and root N content than AB. Compared to PB and PF, PR and AB usually exhibited higher specific root length (SRL) and smaller seed mass.

**Table 1:** Plant functional traits of four functional groups across three vegetation types in the Xilin River Basin of Inner Mongolia grassland.

	Whole plant traits				Leaf traits						Root traits			Reproductive traits	
PFGs	Plant height (cm)	Individual biomass (g)	RSR (g·g^−1^)	SLR (g·g^−1^)	Leaf N (%)	Leaf C:N ratio (g·g^−1^)	SLA (cm^2^·g^−1^)	Pn (µmol CO_2_·m^−2^·s^−1^)	PNUE (µmol CO_2_·mol^−1^·s^−1^)	*δ* ^13^C (‰)	Root N (%)	Root C:N ratio (g·g^−1^)	SRL (m·g^−1^)	Seed mass (mg·seed^−1^)	RA (%)
PB	42.42a	1.99a	0.66ab	2.50a	2.26b	20.96a	93.62b	14.42ab	120.96	−24.40	1.11a	38.17b	97.63	3.57	6.30b
PR	34.28ab	0.56ab	0.28b	0.87b	2.59ab	18.63ab	59.59b	16.50ab	104.04	−26.41	1.01a	45.20b	122.55	1.89	0.25c*
PF	32.72b	1.81a	1.52a	1.33b	2.32b	20.63a	114.21ab	9.80b	68.02	−26.45	1.12a	40.90b	95.54	3.51	12.07b
AB	18.91c	0.48b	0.49b	1.21b	2.79a	17.49b	133.64a	20.35a	139.74	−23.63	0.75b	67.62a	104.10	0.70	24.70a
*P* value	<0.001	0.044	0.018	0.025	0.021	0.092	0.009	0.112	0.182	0.19	<0.001	<0.001	0.846	0.333	0.005

Abbreviations: PFGs, plant functional groups; PB, perennial bunchgrasses; PR, perennial rhizomatous grasses; PF, perennial forbs; AB, annuals and biennials; RSR, root: shoot biomass ratio; SLR, stem: leaf biomass ratio; SLA, specific leaf area; Pn, net photosynthetic rate; PNUE, photosynthetic nutrient use efficiency; δ^13^C, Carbon isotope ratio; SRL, specific root length; RA, reproductive allocation. *, The dominant dispersal mode for perennial rhizomatous grass (i.e., *Leymus chinensis*) is rhizome, with rhizome biomass accounting for 30% of the total plant biomass. *P* values following one-way ANOVAs indicate differences in plant functional traits among four functional groups. Different lowercases represent significant differences among plant functional groups (LSD multiple-range tests, *P*<0.05). The sample sizes of functional traits (plant height, individual biomass, SLR, SLA, leaf N and C: N ratio, and root N and C: N ratio) are 27, 5, 104 and 23 for PB, PR, PF and AB, respectively across meadow, meadow steppe and typical steppes. The sample sizes of functional traits (RSR, Pn, PNUE, δ^13^C, SRL, seed mass and RA) are 5, 3, 10–21 and 8 for PB, PR, PF and AB, respectively in typical steppe.

## Discussion

### Hierarchical Plant C: N: P Stoichiometric Responses to Grazing

Our findings suggest that the effects of grazing on plant C, N, and P contents and stoichiometry increased progressively along the hierarchy of organizational levels, i.e., from plant species to functional group to vegetation type. Grazing enhanced vegetation-type-level N contents characterized by the increased N contents and decreased C: N ratios of plant tissues (leaves and roots) in the Xilin River Basin. However, grazing has contrasting effects on plant stoichiometry and linkage to resource allocation across the three vegetation types, as indicated by the changes in N and P contents, C: N and C: P ratios of leaves and roots at different hierarchical levels (species, functional group, and vegetation type level). In the meadow, grazing enhanced leaf N and P contents but reduced C: N and C: P ratios at three levels of hierarchy. The magnitudes of positive effects of grazing on leaf N and P contents and negative effects on C: N and C: P ratios increased along the hierarchy of organizational levels. In contrast, the magnitude of reduction in leaf N content and increase in C: N ratio diminished considerably along the hierarchy of organizational levels in the typical and meadow steppes. Compared to the leaf stoichiometry, root C: N: P stoichiometry exhibited opposite response patterns to grazing. In the typical and meadow steppes, the magnitudes of positive effects of grazing on root N and P contents and negative effects on C: N and C: P ratios increased along hierarchical levels. In the meadow, however, the magnitude of root stoichiometric responses to grazing decreased at higher hierarchical levels. These findings support our original hypothesis and further suggest that plant stoichiometric responses to grazing are largely mediated by site conditions and are often scale-dependent. Greater and positive leaf responses (i.e., leaf N and P contents increased) but weaker root responses were found in wetter habitats (meadow). On the contrary, stronger and positive root responses (i.e., root N and P contents increased) but weaker and negative leaf responses were found in drier habitats (typical and meadow steppes). This indicates that grazing increases resource allocation to aboveground tissues in the meadow, but it enhances N and P allocation to belowground tissues in the typical and meadow steppes. These results are consistent with the prediction of our second hypothesis, and are also corroborated by previous studies [9], [43]. The minor shifts in soil C, N, and P contents and stoichiometry across the three vegetation types indicate that soil responses to grazing may be subject to a time-lag effect compared to the strong vegetation responses [49], [50].

Our results showed the alterations of C, N and P pools in above- and belowground biomass were caused by changes both in absolute biomass and tissue nutrient contents (leaves and roots). For example, grazing diminished the C, N and P pools of aboveground biomass in the typical steppe, which is mainly attributable to sharply decrease in leaf biomass (by 47%). Grazing enhanced N and P pools of belowground biomass, caused by increases in root N and P contents. The increments in aboveground N and P pools are also mainly attributable to the increased leaf N and P contents in the meadow. In the meadow steppe, however, the increments in belowground C, N and P pools are due to both the increased root biomass (by 71%) and N and P contents. This indicates that the shifts in resource allocation between above- and belowground biomass in response to grazing may change with vegetation type or site conditions. The C, N and P pools of topsoil (0–20 cm) were less affected by grazing across the three vegetation types, because both soil bulk density and nutrient contents remained relatively unchanged. This is consistent with the results from a 7-yr field experiment conducted in the N-limited Minnesota oak savanna [9]. Compared to the meadow steppe and typical steppe, the meadow had much higher above- and belowground standing biomass, and C, N and P pools of above- and belowground biomass and soil. The minor shifts in these plant and soil properties in the Xilin River Basin are attributable to the counterbalance between the positive and negative responses to grazing among vegetation types. This implies that ecosystem resilience to grazing is relatively high in these grasslands, although they have been subjected to free-grazing for 20–30 years. Our findings further suggest that grazing effects on C: N: P stoichiometry are closely linked to ecosystem functions, such as resource allocation and nutrient pools.

### Mechanisms Underpinning Grazing-induced Changes in Plant C: N: P Stoichiometry

Several mechanisms are likely to be responsible for the differential effects of grazing on plant C: N: P stoichiometry across different vegetation types. First, soil properties, particularly soil water and nutrient availability, are likely to be responsible for the contrasting effects of grazing on plant stoichiometry between the meadow and typical steppe. The field holding capacity in the meadow (52%) is two times higher than that in the typical steppe (26%), which may facilitate the utilization of urine and dung deposited by herbivores and improve the soil available N directly, thereby promote plant nutrient absorption [3], [19]. In the typical steppe, however, the low soil moisture and high temperature in the growing season might inhibit the process of N mineralization and nutrient utilization by plants [51]. This is because water availability is a key limiting factor in arid and semiarid grasslands, which is tightly coupled with soil N availability to affect plant growth and primary productivity [42], [45]. In addition, soil carbon and nutrients (N and P) and cation exchange capacity (CEC) are much higher, but soil pH and bulk density are lower in the meadow than the typical steppe [43]. These factors favor tolerant species in the meadow to take up nutrients quickly and accelerate tissue regrowth to compensate for biomass loss by herbivores [9], [35]. Young tissues usually have higher nutrient contents than mature or senescent ones. In the meadow steppe, soil water and nutrient availability are intermediate between the meadow and typical steppe, resulting in a moderate response of plant stoichiometry to grazing. Our findings suggest that the effects of grazing on plant C, N, and P contents and stoichiometry are likely mediated by resource availability. These results are consistent with our original predictions that grazing generally has positive effects on leaf nutrients (e.g., N and P contents) in wet and fertile habitats but had no effects or even negative effects on leaf nutrients in dry and infertile habitats.

Second, plant functional traits, which are closely linked to plant resource-use strategies, above- vs. belowground competitive abilities, and tolerance vs. defense strategies to grazing, are responsible for the diverse stoichiometric responses of different functional groups. The annuals and biennials exhibit acquisitive resource-use strategy in fertile habitats, such as high leaf N content and specific leaf area (SLA), high specific root length (SRL) and root C: N ratio, high net photosynthetic rate (Pn), photosynthetic nutrient use efficiency (PNUE), and water use efficiency (*δ* ^13^C), but low root N content, root: shoot ratio, and leaf C: N ratio, corresponding to great aboveground/light competitive ability. These species also adopt tolerance strategy to grazing, reflecting by high foliar palatability (e.g., high leaf N content, but low leaf C: N ratio) to improve herbivore selectivity, high biomass allocation to leaves (e.g., high leaf N content and SLA, but low stem: leaf ratio, root: shoot ratio, and root N content) to accelerate shoot regrowth following defoliation. Thus annuals and biennials generally allocate more resource to shoots and increase leaf nutrients in response to grazing. In addition, these annuals and biennials have high recruitment capacity, reflected by reproductive traits of small seed, high reproductive output, and short life history. Hence, these species have the competitive advantage in resource exploitation and they maintain relatively stable species compositions across three vegetation types as indicated by their relative biomass.

In contrast, most perennial bunchgrasses are xerophyte which adopt conservative resource-use strategy in dry and nutrient-poor habitats. These species generally exhibit traits characterized by high root N content, root: shoot ratio, and leaf C: N ratio, but low leaf N content, SLA, and SRL, corresponding to great belowground competitive ability. The greater plant height, individual biomass and stem: leaf ratio of these species suggest the functional advantage in light interception, thus they can maintain relatively higher light, nitrogen and water use efficiencies, as reflected by the high Pn, PNUE and *δ* ^13^C. In addition, perennial bunchgrasses adopt defense strategy to grazing, indicated by low foliar palatability (e.g., low leaf N content and SLA, but high leaf C: N ratio) to decrease herbivore selectivity and thereby restrain compensatory growth. Therefore, the perennial bunchgrass dominated plant communities have the advantage in belowground resource competition, leading to weak leaf stoichiometric responses to grazing.

Perennial rhizomatous grasses (i.e., *Leymus chinensis*) are meso-xerophyte which generally adopt both acquisitive and conservative resource-use strategies characterized by relatively higher plant height, leaf N content, and photosynthetic rate (Pn) for aboveground/light competition, and higher root N content and specific root length (SRL) for belowground competition. In addition, these species have high foliar palatability and allocate more biomass to leaves thus exhibiting more tolerance following grazing. Moreover, *L. chinensis* has well-developed laterally spread rhizomes to reproduce vegetative tillers, of which rhizome biomass accounted for 30% of the total plant biomass. The perennial forbs are dominant species in the meadow and meadow steppe and are also widely distributed in the typical steppe. The traits of perennial forbs associated with resource-use strategy, above- vs. belowground competitive ability, and grazing-tolerance are intermediate between the annuals and biennials and perennial bunchgrasses, leading to a moderate response to grazing. The differences in functional traits among different functional groups indicate the fundamental trade-offs between productivity and persistence of species, and further reflect the contrasting species-specific tolerance and defense strategies to grazing. In the Inner Mongolia grassland, the meadow is mainly dominated by perennial forbs and annuals and biennials, with more acquisitive resource-use strategies, great aboveground competitive ability, and fast regrowth following defoliation, suggesting a potential for increasing resource allocation to aboveground tissue and leaf nutrient contents as response to grazing. The typical steppe, however, is dominated by perennial bunchgrasses, with more conservative resource-use strategies, strong belowground competitive ability, and low compensatory growth, indicating a shift towards increasing belowground allocation and root nutrient contents in response to grazing. In the meadow steppe, perennial forbs and perennial bunchgrasses are two dominant life forms, which drive an intermediate response to grazing between the meadow and typical steppe. These results are consistent with the prediction of our second hypothesis that grazing enhances N and P allocation to aboveground in the meadow, but it increases N and P allocation to belowground in the typical and meadow steppes.

Third, the shifts in plant functional group compositions are the major mechanisms driving vegetation-type-level stoichiometric responses and nutrient dynamics. In the meadow, the shift in dominance from perennial bunchgrasses to perennial forbs leads to increase in leaf nutrients (N and P) but no change in root nutrients in response to grazing. On the contrary, the shift in dominance from perennial rhizomatous grasses to perennial bunchgrasses results in the increases in root nutrients (N and P) but no change or even decreases in leaf nutrients in response to grazing. In the meadow steppe, perennial forbs were replaced by perennial rhizomatous grasses and perennial bunchgrasses, leading to an intermediate response between the meadow and typical steppe.

### N Versus P Limitation Across Vegetation Types

Our results suggest that grazing potentially increases P limitation in the semiarid grassland of the Xilin River Basin, as evidenced by the enhanced N content and N: P ratio. The N: P ratio was, on average, 17.2 at the grazed sites, which is higher than the threshold of 16∶1 for P limitation [31]. In addition, the average C: P ratio was 311 at grazed sites, which is higher than the hypothesized threshold of 250∶1 required for efficient growth of P-rich herbivores feeding on comparably C-rich plants, indicating potential P deficiency in these habitats [32]. However, the effects of grazing on nutrient limitation differed across the three vegetation types. Neither N nor P limitation was observed in the meadow, N and P co-limitation in typical steppe, and more P limitation in the meadow steppe. In the meadow, soil nutrients, N and P contents of leaves and roots, and community biomass of above- and belowground were generally higher than those in the typical steppe and meadow steppe. Grazing decreased C: P ratio from 253 to 168 (lower than 250∶1), while N: P ratio (11.4) remained unchanged, which suggests the community is not subject to either N or P limitation. In the typical steppe, both the N: P (19.7) and C: P ratios (345) were relatively less affected by grazing. These factors together with the lowest soil N availability, above- and belowground biomass indicate that the typical steppe is co-limited by N and P. In the meadow steppe, however, grazing enhanced N: P (20.3) and C: P (418) ratios, indicating a shift towards P limitation. In this study, the mean N: P ratio of meadow (12.7 for ungrazed vs. 11.4 for grazed) is lower than previously documented threshold of 14∶1 for N limitation, and N: P ratio of typical steppe (19.6 for ungrazed vs. 19.7 for grazed) is out of the range between 14 and 16 for N and P co-limitation [31], indicating that using foliar N: P ratio to determine nutrient limitation needs more careful interpretation. The limitation threshold may be ecosystem-specific or dependent on site conditions.

In the meadow, grazing enhanced leaf N and P contents, but had little effects on root N and P contents across different hierarchical levels. This indicates more nutrients are reallocated to aboveground for supporting shoot regrowth, and P availability maybe sufficient to support stoichiometrically balanced growth for plants. Our results are corroborated by a previous study [52], which demonstrated that when soil nutrients are abundant, plant nutrient contents (especially P) may be unchanged or even increase following defoliation. It is likely because plants need to allocate sufficient P to recover from defoliation in order to produce ribosomal RNA to replace the N-rich photosynthetic proteins lost to grazers [32], [53]. On the contrary, the enhanced root N and P contents but the unchanged leaf N and declined leaf P content observed in the typical steppe and meadow steppe, suggesting more nutrients are invested to belowground. Previous studies also found grazing-tolerant species exhibit positive feedbacks to herbivory by promoting root exudation of carbon to stimulate rhizospheric microbial populations for soil N mineralization [26], [36]. In our study, we found the dominant perennial bunchgrasses, such as *S. grandis*, *A. cristatum*, *Achnatherum sibiricum*, *C. squarrosa*, and *L. chinensis* in typical steppe generally have roots with rhizosheath, which favors nutrient absorption and water reservation in resource-poor habitats [54]. Tian et al. [55] reported most of dominant and subdominant plant species in typical steppe are colonized by arbuscular mycorrhizal fungi, which could improve plant P uptake, thereby lessen the defoliation-induced P limitation [56]. Nevertheless, these compensatory feedbacks may be insufficient to prevent widespread N or P limitation across a broad geographic region, especially in arid and semiarid grasslands.

### Conclusions

Using a multi-organization-level approach, our study demonstrates the effects of grazing on the C, N and P contents and stoichiometry of plant tissues (leaves and roots) are scale-dependant, and they may change with vegetation type or site conditions. However, soil nutrients exhibit weak responses. Grazing-induced shifts in resource allocation between above- and belowground and nutrient limitation as indicated by leaf stoichiometry differed across vegetation types. Grazing increases N and P allocation to aboveground in the meadow, but it enhances N and P allocation to belowground in the typical and meadow steppes. Neither N nor P limitation was found in the meadow, with N and P co-limitation in the typical steppe and P limitation in the meadow steppe. Our findings suggest that the enhanced vegetation-type-level N contents by grazing and species compensatory feedbacks may be insufficient to prevent widespread declines in primary productivity and nutrient pools in the Inner Mongolia grassland, although they could moderately mitigate the impacts of nutrient limitation. Hence, it is essential to reduce the currently high stocking rates and restore the vast degraded steppes for sustainable development of arid and semiarid grasslands.

## Supporting Information

Figure S1 **Effects of grazing on root C, N, P contents and stoichiometory of dominant species across three vegetation types.** The error bars are mean+SE. Significant differences between the grazed and ungrazed sites are reported from ANOVA as +, 0.05<*P*<0.1; *, *P*<0.05; **, *P*<0.01; ***, *P*<0.001. Abbreviations: Ag, *Agrostis gigantea*; Ca, *Carex appendiculata*; Br, *Blysmus rufus*; Pan, *Potentilla anserina*; Ib, *Inula britanica*; Ss, *Sium suave*; Sb, *Stipa baicalensis*; Ac, *Agropyron cristatum*; Sc, *Serratula centauroides*; As, *Allium senescens*; Pac, *Potentilla acaulis*; Af, *Artemisia frigida*; Lc, *Leymus chinensis*; Sg, *Stipa grandis*; Cs, *Cleistogenes squarrosa*; Ks, *Koeleria cristata*; Cm, *Caragana microphylla*; M, meadow; MS, meadow steppe; TS, typical steppe.
(TIF)

Figure S2 **Effects of grazing on soil C, N, P contents, inorganic N (NH_4_^+^–N and NO_3_^−^–N) and stoichiometory across three vegetation types.** The error bars are mean+SE. M, meadow; MS, meadow steppe; TS, typical steppe; and XRB, Xilin River Basin. Significant differences between the grazed and ungrazed sites are reported from ANOVA as +, 0.05<*P*<0.1; *, *P*<0.05; **, *P*<0.01.
(TIF)

## References

[1] Sasaki T, Okayasu T, Jamsran U, Takeuchi K (2008) Threshold changes in vegetation along a grazing gradient in Mongolian rangelands. Journal of Ecology 96: 145–154.

[2] Frank DA (2005) The interactive effects of grazing ungulates and aboveground production on grassland diversity. Oecologia 143: 629–634. 15800752 10.1007/s00442-005-0019-2

[3] Frank DA, Evans RD (1997) Effects of native grazers on grassland N cycling in Yellowstone National Park. Ecology 78: 2238–2248.

[4] McNaughton SJ, Banyikwa FF, McNaughton MM (1997) Promotion of the cycling of diet-enhancing nutrients by African grazers. Science 278: 1798–1800. 9388182 10.1126/science.278.5344.1798

[5] Semmartin M, Aguiar MR, Distel RA, Moretto AS, Ghersa CM (2004) Litter quality and nutrient cycling affected by grazing-induced species replacements along a precipitation gradient. Oikos 107: 148–160.

[6] Pastor J, Cohen Y, Hobbs T (2006) The role of large herbivores in ecosystem nutrient cycles. In: Danell K, Bergström R, Duncan P, Pastor J, eds. Large mammalian herbivores, ecosystem dynamics, and conservation: Cambridge University Press. 289–325.

[7] Franzluebbers AJ, Stuedemann JA, Schomberg HH, Wilkinson SR (2000) Soil organic C and N pools under long-term pasture management in the Southern Piedmont USA. Soil Biology and Biochemistry 32: 469–478.

[8] Golluscio RA, Austin AT, Martínez GCG, Gonzalez-Polo M, Sala OE, et al (2009) Sheep grazing decreases organic carbon and nitrogen pools in the Patagonian steppe: Combination of direct and indirect effects. Ecosystems 12: 686–697.

[9] Ritchie ME, Tilman D, Knops JMH (1998) Herbivore effects on plant and nitrogen dynamics in oak savanna. Ecology 79: 165–177.

[10] Bagchi S, Ritchie ME (2010) Introduced grazers can restrict potential soil carbon sequestration through impacts on plant community composition. Ecology Letters 13: 959–968. 20482575 10.1111/j.1461-0248.2010.01486.x

[11] Wu HH, Dannenmann M, Fanselow N, Wolf B, Yao ZS, et al (2011) Feedback of grazing on gross rates of N mineralization and inorganic N partitioning in steppe soils of Inner Mongolia. Plant and Soil 340: 127–139.

[12] White R, Murray S, Rohweder M (2000) Pilot analysis of global ecosystems: grassland ecosystems. Washington D.C.: World Resources Institute.

[13] Holland EA, Detling JK (1990) Plant-response to herbivory and belowground nitrogen cycling. Ecology 71: 1040–1049.

[14] Frank DA, Groffman PM (1998) Ungulate vs. landscape control of soil C and N processes in grasslands of Yellowstone National Park. Ecology 79: 2229–2241.

[15] McNaughton SJ (1985) Ecology of a grazing ecosystem: the Serengeti. Ecological Monographs 55: 259–294.

[16] Bryant JP, Provenza FD, Pastor J, Reichardt PB, Clausen TP, et al (1991) Interactions between woody-plants and browsing mammals mediated by secondary metabolites. Annual Review of Ecology and Systematics 22: 431–446.

[17] Pastor J, Naiman RJ (1992) Selective foraging and ecosystem processes in boreal forests. American Naturalist 139: 690–705.

[18] Bardgett RD, Wardle DA (2003) Herbivore-mediated linkages between aboveground and belowground communities. Ecology 84: 2258–2268.

[19] Augustine DJ, McNaughton SJ (2006) Interactive effects of ungulate herbivores, soil fertility, and variable rainfall on ecosystem processes in a semi-arid savanna. Ecosystems 9: 1242–1256.

[20] Wardle DA, Bardgett RD, Klironomos JN, Setala H, van der Putten WH, et al (2004) Ecological linkages between aboveground and belowground biota. Science 304: 1629–1633. 15192218 10.1126/science.1094875

[21] Díaz S, Hodgson JG, Thompson K, Cabido M, Cornelissen JHC, et al (2004) The plant traits that drive ecosystems: Evidence from three continents. Journal of Vegetation Science 15: 295–304.

[22] Díaz S, Lavorel S, de Bello F, Quetier F, Grigulis K, et al (2007) Incorporating plant functional diversity effects in ecosystem service assessments. Proceedings of the National Academy of Sciences of the United States of America 104: 20684–20689. 18093933 10.1073/pnas.0704716104 PMC2410063

[23] Díaz S, Lavorel S, McIntyre S, Falczuk V, Casanoves F, et al (2007) Plant trait responses to grazing - a global synthesis. Global Change Biology 13: 313–341.

[24] Elser JJ, Fagan WF, Kerkhoff AJ, Swenson NG, Enquist BJ (2010) Biological stoichiometry of plant production: metabolism, scaling and ecological response to global change. New Phytologist 186: 593–608. 20298486 10.1111/j.1469-8137.2010.03214.x

[25] Holland JN, Cheng WX, Crossley DA (1996) Herbivore-induced changes in plant carbon allocation: assessment of below-ground C fluxes using carbon-14. Oecologia 107: 87–94. 28307195 10.1007/BF00582238

[26] Bardgett RD, Wardle DA, Yeates GW (1998) Linking above-ground and below-ground interactions: how plant responses to foliar herbivory influence soil organisms. Soil Biology and Biochemistry 30: 1867–1878.

[27] Shan YM, Chen DM, Guan XX, Zheng SX, Chen HJ, et al (2011) Seasonally dependent impacts of grazing on soil nitrogen mineralization and linkages to ecosystem functioning in Inner Mongolia grassland. Soil Biology and Biochemistry 43: 1943–1954.

[28] Tilman D (1988) Plant strategies and the dynamics and structure of plant communities. Princeton, New Jersey: Princeton University Press.

[29] Poorter H, Nagel O (2000) The role of biomass allocation in the growth response of plants to different levels of light, CO_2_, nutrients and water: a quantitative review. Australian Journal of Plant Physiology 27: 595–607.

[30] Pan Q, Bai Y, Wu J, Han X (2011) Hierarchical plant responses and diversity loss after nitrogen addition: Testing three functionally-based hypotheses in the Inner Mongolia grassland. PLoS ONE 6: e20078. 21625503 10.1371/journal.pone.0020078 PMC3098266

[31] Koerselman W, Meuleman AFM (1996) The vegetation N:P ratio: a new tool to detect the nature of nutrient limitation. Journal of Applied Ecology 33: 1441–1450.

[32] Elser JJ, Fagan WF, Denno RF, Dobberfuhl DR, Folarin A, et al (2000) Nutritional constraints in terrestrial and freshwater food webs. Nature 408: 578–580. 11117743 10.1038/35046058

[33] Gusewell S (2004) N : P ratios in terrestrial plants: variation and functional significance. New Phytologist 164: 243–266. 10.1111/j.1469-8137.2004.01192.x 33873556

[34] Aerts R (1997) Climate, leaf litter chemistry and leaf litter decomposition in terrestrial ecosystems: a triangular relationship. Oikos 79: 439–449.

[35] Hobbie SE (1992) Effects of plant-species on nutrient cycling. Trends in Ecology and Evolution 7: 336–339. 21236058 10.1016/0169-5347(92)90126-V

[36] Hamilton EW III, Frank DA (2001) Can plants stimulate soil microbes and their own nutrient supply? Evidence from a grazing tolerant grass. Ecology 82: 2397–2402.

[37] Zheng SX, Lan ZC, Li WH, Shao RX, Shan YM, et al (2011) Differential responses of plant functional trait to grazing between two contrasting dominant C_3_ and C_4_ species in a typical steppe of Inner Mongolia, China. Plant and Soil 340: 141–155.

[38] Craine JM, Froehle J, Tilman DG, Wedin DA, Chapin FS (2001) The relationships among root and leaf traits of 76 grassland species and relative abundance along fertility and disturbance gradients. Oikos 93: 274–285.

[39] Cornelissen JHC, Lavorel S, Garnier E, Díaz S, Buchmann N, et al (2003) A handbook of protocols for standardised and easy measurement of plant functional traits worldwide. Australian Journal of Botany 51: 335–380.

[40] Naeem S (1998) Species redundancy and ecosystem reliability. Conservation Biology 12: 39–45.

[41] Loreau M, Hector A (2001) Partitioning selection and complementarity in biodiversity experiments. Nature 412: 72–76. 11452308 10.1038/35083573

[42] Bai YF, Han XG, Wu JG, Chen ZZ, Li LH (2004) Ecosystem stability and compensatory effects in the Inner Mongolia grassland. Nature 431: 181–184. 15356630 10.1038/nature02850

[43] Zheng SX, Ren HY, Lan ZC, Li WH, Wang KB, et al (2010) Effects of grazing on leaf traits and ecosystem functioning in Inner Mongolia grasslands: scaling from species to community. Biogeosciences 7: 1117–1132.

[44] Jiang GM, Han XG, Wu JG (2006) Restoration and management of the Inner Mongolia Grassland require a sustainable strategy. AMBIO 35: 269–270. 16989513 10.1579/06-s-158.1

[45] Bai YF, Wu JG, Xing Q, Pan QM, Huang JH, et al (2008) Primary production and rain use efficiency across a precipitation gradient on the Mongolia plateau. Ecology 89: 2140–2153. 18724724 10.1890/07-0992.1

[46] Bai YF, Li LH, Wang QB, Zhang LX, Zhang Y, et al (2000) Changes in plant species diversity and productivity along gradients of precipitation and elevation in the Xilin River Basin, Inner Mongolia. Acta Phytoecologica Sinica 24: 667–673.

[47] Sparks DL, Page AL, Helmke PA, Loeppert RH, Soltanpour PN, et al.. (1996) Methods of soil analysis Part 3: Chemical methods. Madison, WI, USA: Soil Science Society of America, Inc. and American Society of Agronomy, Inc.

[48] Pregitzer KS, DeForest JL, Burton AJ, Allen MF, Ruess RW, et al (2002) Fine root architecture of nine North American trees. Ecological Monographs 72: 293–309.

[49] Milchunas DG, Lauenroth WK (1993) Quantitative effects of grazing on vegetation and soils over a global range of environments. Ecological Monographs 63: 327–366.

[50] Zhou ZY, Li FR, Chen SK, Zhang HR, Li GD (2011) Dynamics of vegetation and soil carbon and nitrogen accumulation over 26 years under controlled grazing in a desert shrubland. Plant and Soil 341: 257–268.

[51] Shan YM, Chen DM, Guan XX, Zheng SX, Chen HJ, et al (2011) Seasonally dependent impacts of grazing on soil nitrogen mineralization and linkages to ecosystem functioning in Inner Mongolia grassland. Soil Biology and Biochemistry 43: 1943–1954.

[52] McNaughton SJ, Chapin FS III (1985) Effects of phosphorus nutrition and defoliation on C_4_ graminoids from the serengeti plains. Ecology 66: 1617–1629.

[53] Matzek V, Vitousek PM (2009) N : P stoichiometry and protein : RNA ratios in vascular plants: an evaluation of the growth-rate hypothesis. Ecology Letters 12: 765–771. 19392715 10.1111/j.1461-0248.2009.01310.x

[54] McCully ME (1999) Roots in soil: unearthing the complexities of roots and their rhizospheres. Annual Review of Plant Physiology and Plant Molecular Biology 50: 695–718. 10.1146/annurev.arplant.50.1.695 15012224

[55] Tian H, Gai JP, Zhang JL, Christie P, Li XL (2009) Arbuscular mycorrhizal fungi associated with wild forage plants in typical steppe of eastern Inner Mongolia. European Journal of Soil Biology 45: 321–327.

[56] van der Heijden MGA, Klironomos JN, Ursic M, Moutoglis P, Streitwolf-Engel R, et al (1998) Mycorrhizal fungal diversity determines plant biodiversity, ecosystem variability and productivity. Nature 396: 69–72.

