# Supplementary figures and images for: Scale-Dependent Effects of Grazing on Plant C: N: P Stoichiometry and Linkages to Ecosystem Functioning in the Inner Mongolia Grassland
Source: PLoS One. 2012 Dec 14;7(12):e51750. doi: 10.1371/journal.pone.0051750 (PMC3522734; doi:10.1371/journal.pone.0051750)

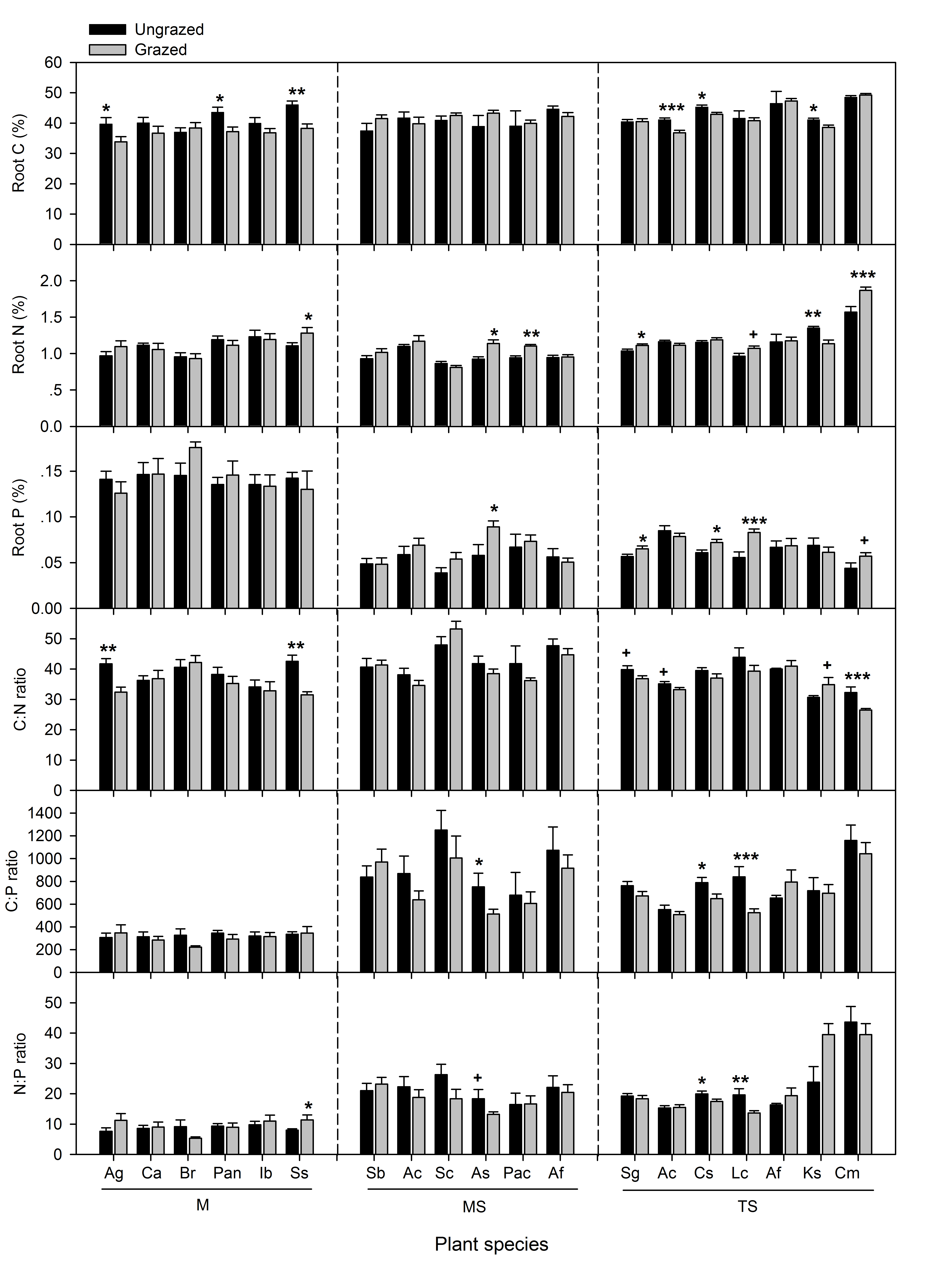

Supplement: Figure S1 — Effects of grazing on root C, N, P contents and stoichiometory of dominant species across three vegetation types. The error bars are mean+SE. Significant differences between the grazed and ungrazed sites are reported from ANOVA as +, 0.05<P<0.1; *, P<0.05; **, P<0.01; ***, P<0.001. Abbreviations: Ag, Agrostis gigantea; Ca, Carex appendiculata; Br, Blysmus rufus; Pan, Potentilla anserina; Ib, Inula britanica; Ss, Sium suave; Sb, Stipa baicalensis; Ac, Agropyron cristatum; Sc, Serratula centauroides; As, Allium senescens; Pac, Potentilla acaulis; Af, Artemisia frigida; Lc, Leymus chinensis; Sg, Stipa grandis; Cs, Cleistogenes squarrosa; Ks, Koeleria cristata; Cm, Caragana microphylla; M, meadow; MS, meadow steppe; TS, typical steppe. (TIF) [file pone.0051750.s001.tif]

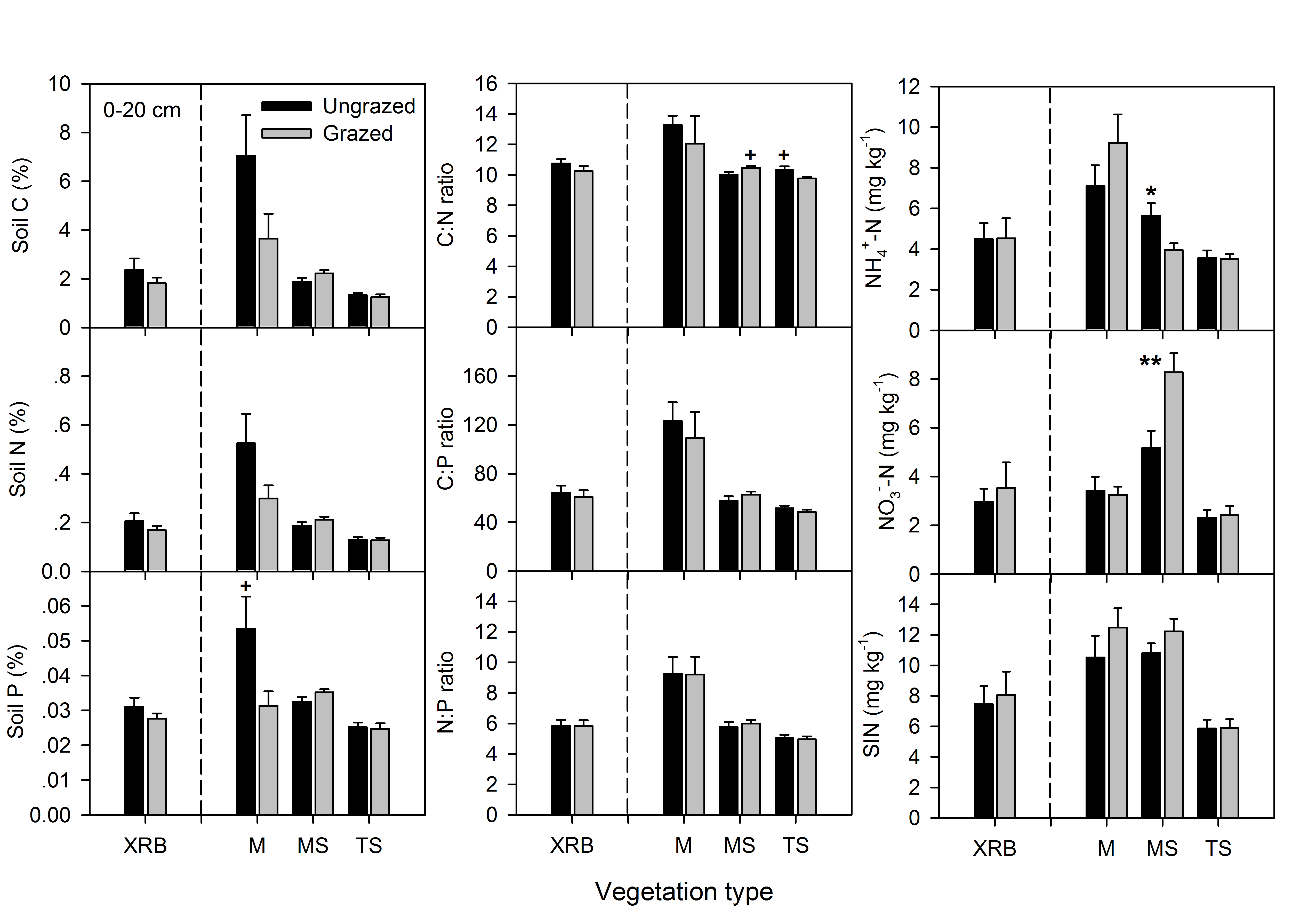

Supplement: Figure S2 — Effects of grazing on soil C, N, P contents, inorganic N (NH4+–N and NO3−–N) and stoichiometory across three vegetation types. The error bars are mean+SE. M, meadow; MS, meadow steppe; TS, typical steppe; and XRB, Xilin River Basin. Significant differences between the grazed and ungrazed sites are reported from ANOVA as +, 0.05<P<0.1; *, P<0.05; **, P<0.01. (TIF) [file pone.0051750.s002.tif]
